# Incorporating a Survivorship Clinic Into Practice

**Published:** 2016-04-01

**Authors:** Denice Economou, Stacie Corcoran

**Affiliations:** City of Hope, Duarte, California, and Memorial Sloan Kettering Cancer Center, New York, New York

The rising number of cancer survivors poses distinct challenges to the health care community. Speakers at JADPRO Live at APSHO addressed the need to improve access to care, quality of life, and health outcomes for survivors. Given their clinical expertise, their ability to practice in numerous settings, and their reputation as leaders in developing and evaluating innovative care models, advanced practitioners (APs) are the ideal candidates to provide follow-up care for cancer survivors.

## OVERVIEW OF SURVIVORSHIP CARE

By 2022 there will be 18 million cancer survivors in the United States (Institute of Medicine, 2013). When broken down by cancer type, prostate cancer is the leader for men and comprises 43% of survivors and breast cancer is the leader for women at 41% (American Cancer Society, 2014). "The number of survivors keeps growing, which means that we’re doing the right thing," said Denice Economou, RN, MN, CHPN, of City of Hope, Duarte, California. "Those of us who are older nurses are very excited and proud of the work we’ve done to lead to this."

At the same time, she added, the growth in survivorship poses challenges for the healthcare community. Patients and providers have increasing expectations for good quality of life after cancer, which can be compromised by the medical consequences of treatment.

"There is a wide range of potential long-term and late effects that persist after completion of treatment, and they are often unanticipated," Ms. Economou observed.

Risk, she explained, depends on the patient’s tumor type and age at the time of treatment, and the type and intensity of treatment. "Some of our pediatric patients deal with issues that go way beyond what we see in adult survivors, which is why we try to run those programs and educate separately," she said.

**Figure 1:**
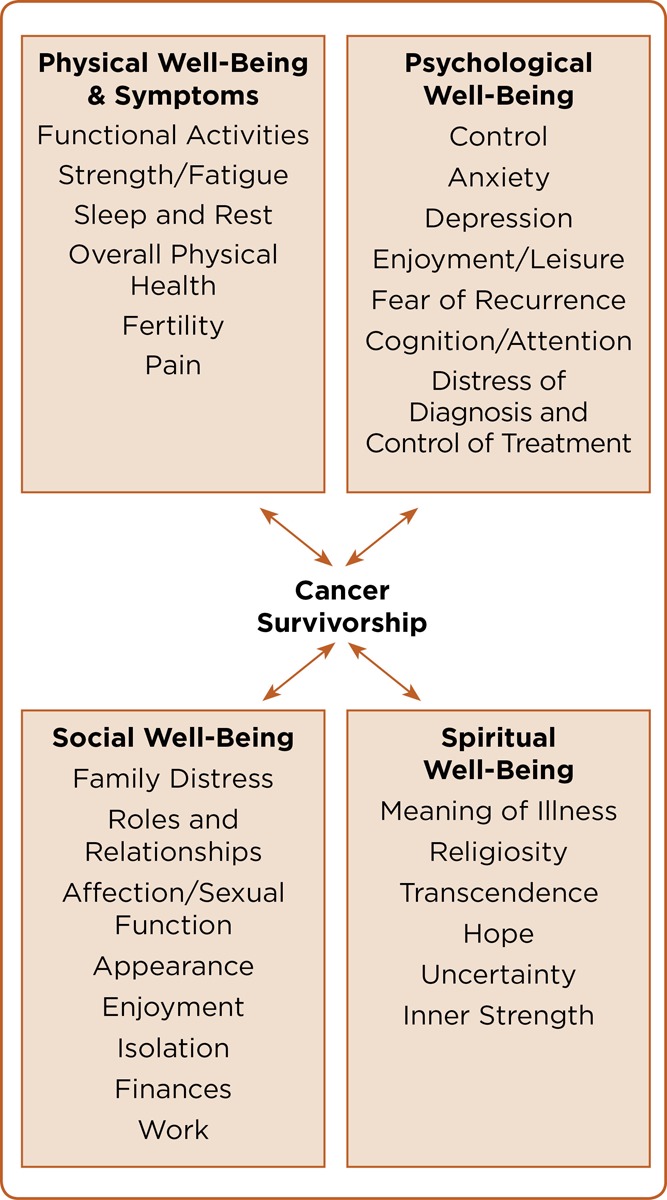
Dimensions of quality of life in cancer survivors. Adapted from Ferrell and Grant (2005)

Cancer survivors are affected physically, psychologically, socially, and spiritually (King et al., 1997), and survivorship care should touch upon all these areas, said Ms. Economou (Figure 1).

## SURVIVORS’ NEEDS

The LIVESTRONG foundation polled more than 1,000 survivors and found the following to be their main concerns: secondary health problems (53%), chronic pain (54%), and infertility (33%; Rechis, Beckjork, & Nutt, 2014). The survey also documented additional concerns:

- Nonmedical support: 49% indicated that nonmedical cancer needs were unmet; 53% felt the practical and emotional consequences of cancer are often more difficult than medical issues.
- Emotional support: 70% were dealing with depression, but only 22% acknowledged seeking professional services.
- Relationships: 58% of survivors reported loss of sexual desire and/or sexual function.
- Financial problems: 43% had decreased income as a result of cancer, 25% were in debt, and 12% turned down a treatment option because of cost.
- Job issues: 32% indicated lack of advancement, demotion, or job loss due to their diagnosis, and 34% felt trapped in a job to preserve insurance coverage.

## SURVIVORSHIP CARE ACROSS THE CONTINUUM

Survivorship is increasingly being defined as the time "from diagnosis to death," which means thinking about survivorship from the start and developing a protocol, Ms. Economou said.

**Figure 2:**
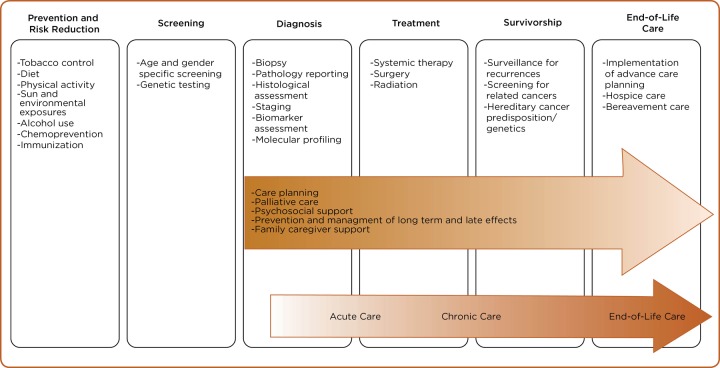
Cancer care continuum. Information from Rechis, Beckjord, Arvey, Reynolds, and McGoldrick (2011).

"We don’t just drop you off the face of the earth once you finish chemotherapy…palliative care is a fabulous resource for all of us. It can provide symptom management for side effects post-treatment and in the long term," she said (Figure 2).

The Commission on Cancer Standard 3.3 Survivorship Care Plan (SCP) requires that all cancer programs provide a summary of treatment and a follow-up plan to all patients completing cancer treatments. By January 1, 2016, 25% of eligible patients should receive an SCP, said Ms. Economou, ramping up to 100% of eligible patients receiving a care plan by 2019 (Commission on Cancer, 2012).

## DEVELOPING AP-LED SURVIVORSHIP CARE

Stacie Corcoran, RN, MS, AOCNS®, of Memorial Sloan Kettering Cancer Center, New York, said survivorship care offers many opportunities for APs, who practice in many different healthcare settings and "who are known providers of quality and cost-effective care."

Ms. Corcoran cited an anticipated shortage of more than 1480 oncologists by 2025, while numbers of APs are increasing.

The first step, said Ms. Corcoran, is collaborating with the medical team to determine what survivor population will be served. "You want to find your champions," she said, "the physicians who are really going to work with you in developing this and are committed to sending patients to the AP clinic."

Next, it is important to understand the components of the survivorship visit, which should include surveillance for recurrence; identification and management of long-term and late effects; health-promotion counseling; communication with primary care providers and others involved in patient care; and the SCP.

"For an AP-led survivorship clinic, you really want great consistency," she said. "You want to have this nailed down before you’ve started."

## MODEL OF CARE SELECTION

A needs assessment can identify strengths and deficits in one’s practice setting, which can help select the best model of care from among these, as described by Ms. Corcoran and explained below.

**Shared-Visit Models**

*Specific or multiple disease model*—In this familiar model, she said, "a physician and an AP collaborate, specializing in one or many diseases, and they divide up their responsibilities."

*Multidisciplinary model*—In this model, which can serve a variety of diagnoses, multiple providers and services are available during one visit. This is typically the oncologist and AP, social worker, and physical therapist. "This is something that’s very resource- and cost-intense," she said, "so it’s not very common in many settings for that reason."

**Independent-Visit Models**

*Consultative model*—The AP provides an independent, one-time visit. With late-effect monitoring, screening recommendations, health promotion, and SCP, this model is survivorship-focused and can serve a variety of diagnoses. "This is a fairly common model," she said.

*Integrated/ongoing care AP model*—This model focuses on a specific diagnosis and may be embedded in the disease/treatment area. The AP independently cares for posttreatment patients with a focus on long-term needs, essentially taking over full oncologic care.

## TRANSITION TO PRIMARY CARE

This is a risk-based approach for patients considered low-risk for late treatment effects and low-risk for disease recurrence and appropriate for transition from oncology specialist to PCP. "This has been done for some time in Canada and England," said Ms. Corcoran, "but we have been slower to adopt this here in the US."

## IMPLEMENTING AP-LED SURVIVORSHIP CARE

Ms. Corcoran acknowledged that it takes time to lay the groundwork for a clinic. Once the model of care is selected, the program scope and resources should be considered. Realistically, this can take 6 months to 1 year.

In selecting APs for the clinic, experience counts, especially if the role will be independent and autonomous. "Having the right degree and certifications obviously helps," she said, "but having that judgment and autonomy that only comes with experience can be very useful."

Staff should be trained in how to communicate the purpose of the survivorship referral to patients. It is helpful to identify eligible patients early so that physicians can make timely referrals. Educational material for patients should be on hand, she added.

Finally, the survivorship care program should be evaluated. Some simple process measures include rate of referral from physician to AP; patient acceptance; patient satisfaction; patient adherence to screening guidelines recommendations; incidence of psychosocial counseling based on distress screening; and SCP delivery rate. Cost metrics include AP revenue, payer reimbursement, and downstream referrals and revenue at the institution.
